# Quantifying the Stability of Coupled Genetic and Epigenetic Switches With Variational Methods

**DOI:** 10.3389/fgene.2020.636724

**Published:** 2021-01-22

**Authors:** Amogh Sood, Bin Zhang

**Affiliations:** Department of Chemistry, Massachusetts Institute of Technology, Cambridge, MA, United States

**Keywords:** gene expression noise, minimum action, chromatin state, gene network, self-regulating gene

## Abstract

The Waddington landscape provides an intuitive metaphor to view development as a ball rolling down the hill, with distinct phenotypes as basins and differentiation pathways as valleys. Since, at a molecular level, cell differentiation arises from interactions among the genes, a mathematical definition for the Waddington landscape can, in principle, be obtained by studying the gene regulatory networks. For eukaryotes, gene regulation is inextricably and intimately linked to histone modifications. However, the impact of such modifications on both landscape topography and stability of attractor states is not fully understood. In this work, we introduced a minimal kinetic model for gene regulation that combines the impact of both histone modifications and transcription factors. We further developed an approximation scheme based on variational principles to solve the corresponding master equation in a second quantized framework. By analyzing the steady-state solutions at various parameter regimes, we found that histone modification kinetics can significantly alter the behavior of a genetic network, resulting in qualitative changes in gene expression profiles. The emerging epigenetic landscape captures the delicate interplay between transcription factors and histone modifications in driving cell-fate decisions.

## 1. Introduction

A little more than five decades ago, Waddington introduced the metaphor to view cellular differentiation into distinct lineages and cell types as a sequence of transitions among basins in a landscape, wherein basins indicate stable phenotypes (Waddington and Kacser, 1957). The appeal of this metaphor to intuition has inspired efforts of theoretical formulation at the molecular level by studying genetic networks formed by transcription factors (TF) (Sasai and Wolynes, 2003; Hornos et al., 2005; Kærn et al., 2005; Walczak et al., 2005a,b; Xu and Tao, 2006; Goldberg et al., 2007; Kim and Wang, 2007; Shahrezaei and Swain, 2008; Cao et al., 2010; Venegas-Ortiz and Evans, 2011; Wang et al., 2011, 2014; Zhang et al., 2013; Zhang and Wolynes, 2014; Lv et al., 2015; Chen et al., 2016; Qiu et al., 2020). These studies highlighted the importance of gene expression noise in driving the transition among steady states. Noise is a manifestation of the inherent stochasticity of chemical reactions and arises in gene regulatory networks as a result of protein production/degradation and TF binding/unbinding. Noise, or fluctuation, is non-negligible due to the finite number of protein molecules and the single molecule nature of DNA. Stochastic noise and network topology together define the epigenetic landscape, much like the one envisioned by Waddington, that quantifies the stability of various cell-defining gene expression levels or patterns.

For eukaryotic organisms, in addition to transcription factors, epigenetic marks such as DNA methylation and histone modifications also play essential roles in regulating gene expression (Lister et al., 2009; Lu et al., 2009; Artyomov et al., 2010; Krishnakumar and Kraus, 2010; Margueron and Reinberg, 2010; Mariani et al., 2010; Andrew Angel, 2011; Miller-Jensen et al., 2011; Furey and Sethupathy, 2013). They are known to affect local chromatin packaging and global genome organization (Zhou et al., 2011; Schlick et al., 2012; Rowley and Corces, 2018; Parsons and Zhang, 2019; Qi et al., 2020; Xie et al., 2020), which in turn can regulate DNA accessibility to regulatory proteins. Furthermore, DNA methylation directly impacts the DNA binding affinity of transcription factors (Tate and Bird, 1993; Zhou et al., 2016; Flavahan et al., 2019). Importantly, the chemical modifications themselves may give rise to steady states independent of the TF-centric genetic network. For example, modification of nucleosomes recruits enzymes affecting the neighboring nucleosomes, causing them to be similarly modified (Bannister and Kouzarides, 2011). Many elegant theoretical attempts have demonstrated how such interactions can bring about collective changes of many nucleosomes and allow them to exhibit distinct multistable states (Dodd et al., 2007; Sedighi and Sengupta, 2007; David-Rus et al., 2009; Micheelsen et al., 2010; Sneppen and Mitarai, 2012; Dayarian and Sengupta, 2013; Jost, 2014; Sood and Zhang, 2020). Therefore, it is crucial to account for the dynamics and regulation of epigenetic modifications when constructing the landscape for cellular differentiation in eukaryotes.

Many research groups have studied the interplay between genetic and epigenetic switches in regulating gene expression. For instance, generalized genetic networks that couple each gene to a binary or ternary variable representing the collective histone states have been used as models for stem cells to account for epigenetic degrees of freedom, albeit in a coarse grained fashion (Artyomov et al., 2010; Binder et al., 2013; Sasai et al., 2013; Ashwin and Sasai, 2015; Huang and Lei, 2018; Folguera-Blasco et al., 2019). These studies found a significant dependence of the probability landscape of protein expression computed from stochastic simulations on chromatin state dynamics. Similarly coarse-grained treatment of epigenetic switches was shown to introduce hysteresis (Bhattacharyya et al., 2020) and homeorhesis (Matsushita and Kaneko, 2020) to the dynamics of gene regulatory networks. Notably, Zhang et al. (2019) explicitly considered the modification of individual nucleosomes and studied the impact of such modifications on the probability landscape of a single self-activating gene and a pair of mutually repressive genes. However, the lack of analytical results has made the sensitivity analysis of the computed landscape with respect to parameter values, which may vary along cell differentiation, numerically challenging.

In this work, we investigate the combined impact of TF binding and epigenetic modifications in regulating the expression of a self-activating gene. Rather than coarse-graining the epigenetic switch into a binary or ternary variable, we explicitly account for the dynamical modification of individual nucleosomes. The variational approach (Eyink, 1996; Sasai and Wolynes, 2003) was used to compute steady-state probability distributions from deterministic equations and avoid computationally intensive stochastic simulations. Moreover, we generalize the typically used Poisson ansatz to better treat systems with particle conservation constraints, such as our epigenetic switch, that are more naturally described using SU(2) than Bosonic operators (Sood and Zhang, 2020). The approach enabled a convenient exploration of the model's steady-state behavior across a wide range of parameters. Our study suggests that fast, random perturbations to individual histone modifications lead to the formation of a poised, uncommitted chromatin state, which in turn can drive noisy gene expression seen in stem cells. As the rate of such random perturbations decreases and the role of cooperative modifications of nucleosome prevails, the system transitions to a bistable regime resembling a differentiated state. The transition goes through an activated state with high gene expression, highlighting the robustness of the network in activating gene expression due to the feedback between genetic and epigenetic switches. We further compared variational results with stochastic simulations and discussed potential improvements in the accuracy of the variational method.

## 2. Model

We consider a simplified model of eukaryotic gene regulation that accounts for TF binding/unbinding as well as histone modifications. The model couples the regulatory network of a self-activating gene with an epigenetic switch that can lead to active and repressive chromatin states.

**Figure 1:**
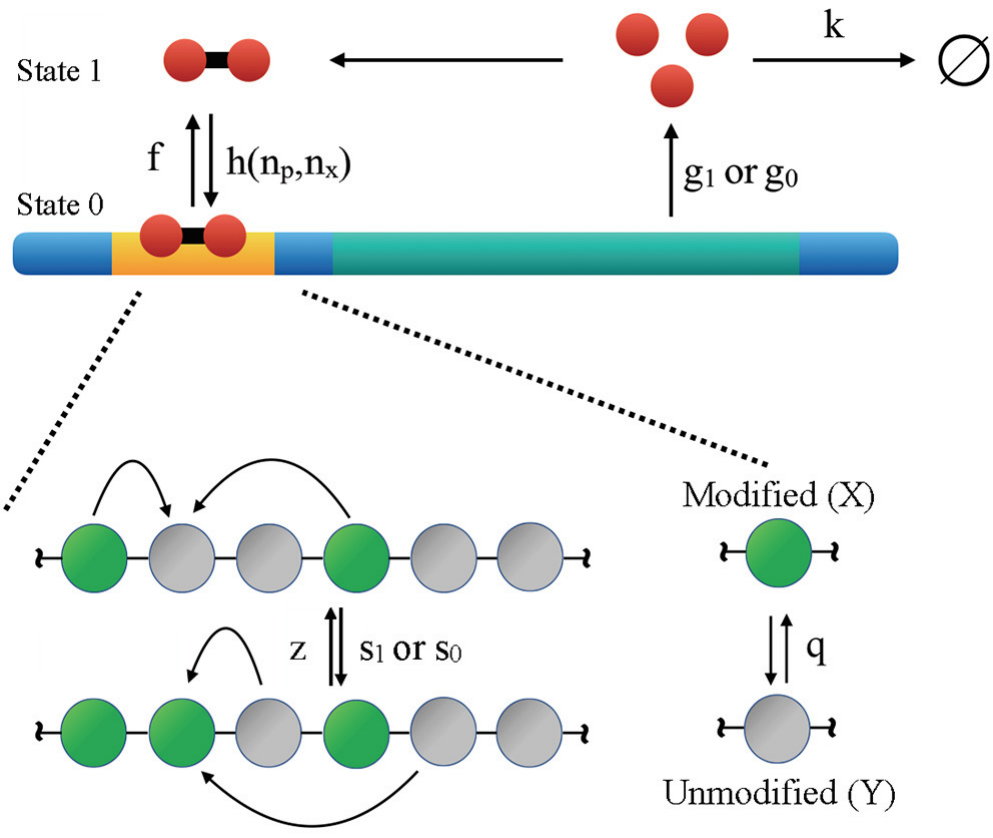
Illustration of the kinetic model that couples the regulatory network of a self-activating gene with the reaction network of histone modifications. The gene is auto-regulatory as the protein produced by the gene (red circles) binds to the promoter region (yellow) with rate *h* and unbinds with rate *f*. Depending on whether the regulatory protein is bound (State 0) or unbound (State 1), the rate of protein production is *g*_0_ or *g*_1_. Proteins degrade with rate *k*. Conversions between modified (X) and unmodified (Y) nucleosomes can occur “randomly” (irrespective to the status of other nucleosomes) with a basal rate *q*. Nucleosome modifications can also occur more cooperatively with rate of *z* and *s*.

For self-activating genes, their protein products bind with the promoter to upregulate the transcription rate. As illustrated in Figure 1, proteins are produced and destroyed with rates of *g* and *k*, respectively. The protein production rate is further dependent on whether the gene's promoter is bound by TF (state 0) or not (state 1), and we have *g*_1_ < *g*_0_ since the proteins are activators. Here TFs correspond to gene transcription products, and they bind to the promoter with rate *h* as dimers. The corresponding unbinding rate is *f*. Binding rate depends on protein copy number *n*_*p*_ as well as the number of modified nucleosomes *n*_*x*_ as detailed in Equation (3) below. Self-activating genes are known to occur both as isolated entities (Ptashne et al., 1980; Johnson et al., 1981; Hasty et al., 2000; Rosenfeld et al., 2002) and as common motifs of larger interacting networks (Ralston and Rossant, 2005; Loh et al., 2008; Orkin and Zon, 2008). They have been the subject of extensive theoretical study as models of cellular differentiation (Sasai and Wolynes, 2003; Hornos et al., 2005; Walczak et al., 2005a,b; Xu and Tao, 2006; Goldberg et al., 2007; Kim and Wang, 2007; Shahrezaei and Swain, 2008; Venegas-Ortiz and Evans, 2011; Wang et al., 2011; Zhang et al., 2013; Zhang and Wolynes, 2014). The epigenetic switch concerns a cluster of *N* = 60 nucleosomes, each of which can exist in a modified (*X*) or unmodified (*Y*) state. The kinetics of chromatin system can be described with the non-linear dynamics given below

(1) $$\begin{array}{l} X+X+Y\overset{s_{1},s_{0}}{\to }3X,\;Y+Y+X\overset{z}{\to }3Y, \end{array}$$

(2) $$\begin{array}{l} X\overset{q}{\to }Y,\;Y\overset{q}{\to }X. \end{array}$$

The inter-conversion between modified and unmodified nucleosomes can either proceed via Equation (1) that requires a pair of similarly modified nucleosomes to alter the state of a nucleosome, or via noisy conversion (Equation 2) with first-order kinetics. The former is meant to account for nucleosomes being actively interconverted by modifying and removing enzymes recruited by the similarly modified nucleosomes in their vicinity. It is this recruitment that forms the positive feedback in the system (Dodd et al., 2007; Micheelsen et al., 2010; Xie and Zhang, 2019; Sood and Zhang, 2020). *s, z*, and *q* are the rate constants of the corresponding reactions.

The coupling between the genetic and epigenetic switch is achieved by introducing a dependence of protein binding rate on the number of modified nucleosomes, i.e.,

(3) $$\begin{array}{l} h\left(n_{p},n_{x}\right)=h_{o}\frac{n_{p}\left(n_{p}-1\right)}{1+\text{exp}\left(-0.5\left(n_{x}-35\right)\right)}. \end{array}$$

This dependence is motivated by the realization that actively modified chromatin (*n*_*x*_ > 35) exists in a more open state that is more accessible to regulatory proteins. The particular expression [1 + exp(−0.5(*n_x_* − 35))]^−1^ as the probability for chromatin being open is typical of a two state system, assuming that the energetic difference between open and closed chromatin depends linearly on the number of modified nucleosomes. Furthermore, the recruited conversion rate of unmodified to modified nucleosomes depends on TF binding with *s*_0_ > *s*_1_, assuming that TFs can attract modification enzymes to chromatin. The values for the kinetic parameters were set relative to the degradation rate *k* as *g*_1_ = 4, *g*_0_ = 65, *h*_*o*_ = 1, *f* = 100, *s*_1_ = 8, *s*_0_ = 10*s*_1_, *z* = 8. The random histone modification rate, *q*, was varied over a wide range of values as detailed below. We used *k* = 1*s*^−1^, though changing this value will not affect the steady state distributions and only renormalizes the timescale in the model.

**Figure 2:**
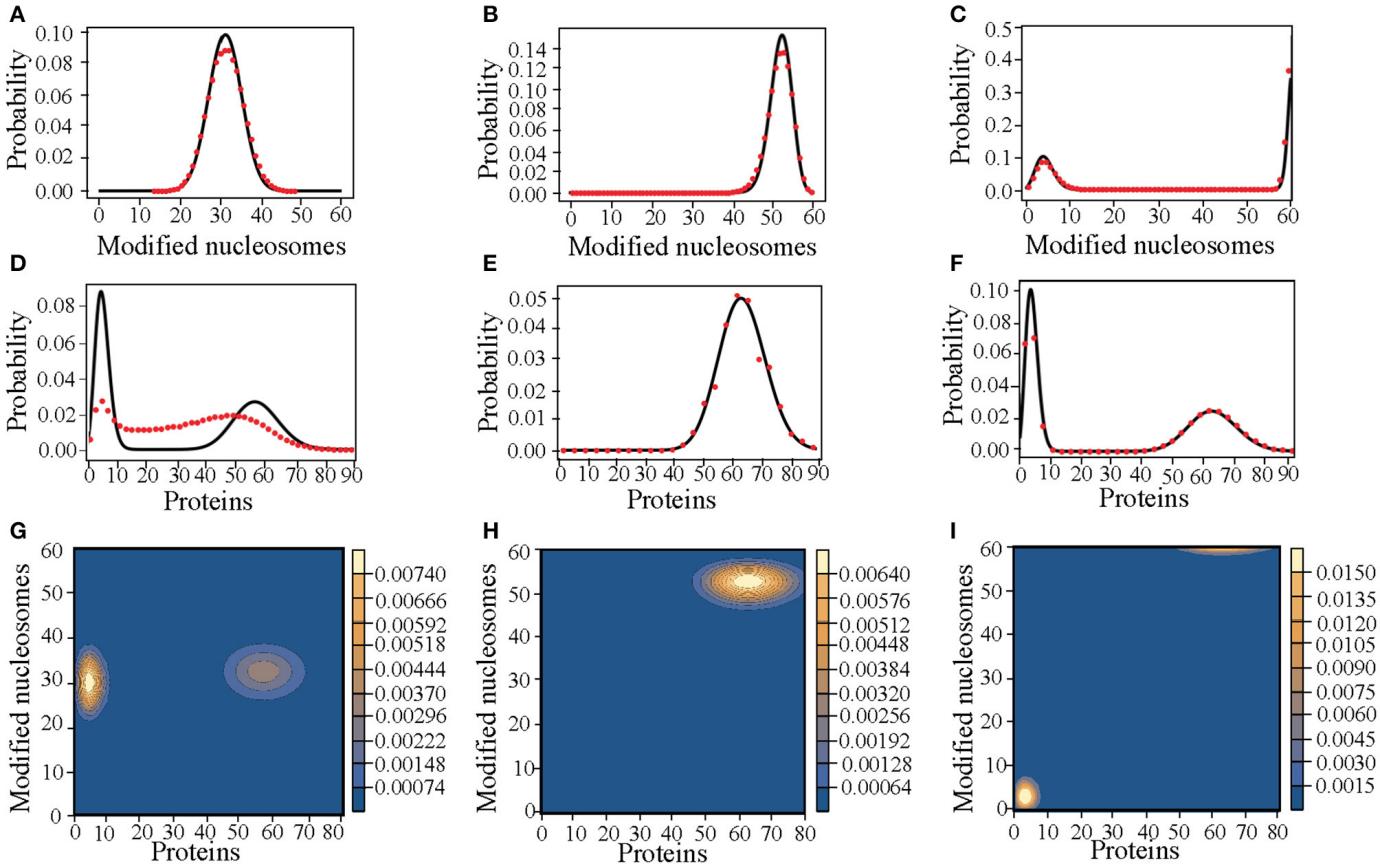
Comparison between the probability distributions obtained from the variational approach and from stochastic simulations. **(A–C)** Steady state probability distributions for the number of modified nucleosomes computed using the variational method (black solid line) and from stochastic simulations (red dots) for *q* = 100 **(A)**, 10 **(B)**, and 0.5 **(C)**. **(D–F)** Steady state probability distributions for the number of protein molecules computed using the variational method (black solid line) and from stochastic simulations (red dots) for *q* = 100 **(D)**, 10 **(E)**, and 0.5 **(F)**. **(G–I)** Steady state probability distributions as a function of both number of proteins and modified nucleosomes computed using the variational method for *q* = 100 **(G)**, 10 **(H)**, and 0.5 **(I)**, showing two, one and two fixed points, respectively.

**Figure 3:**
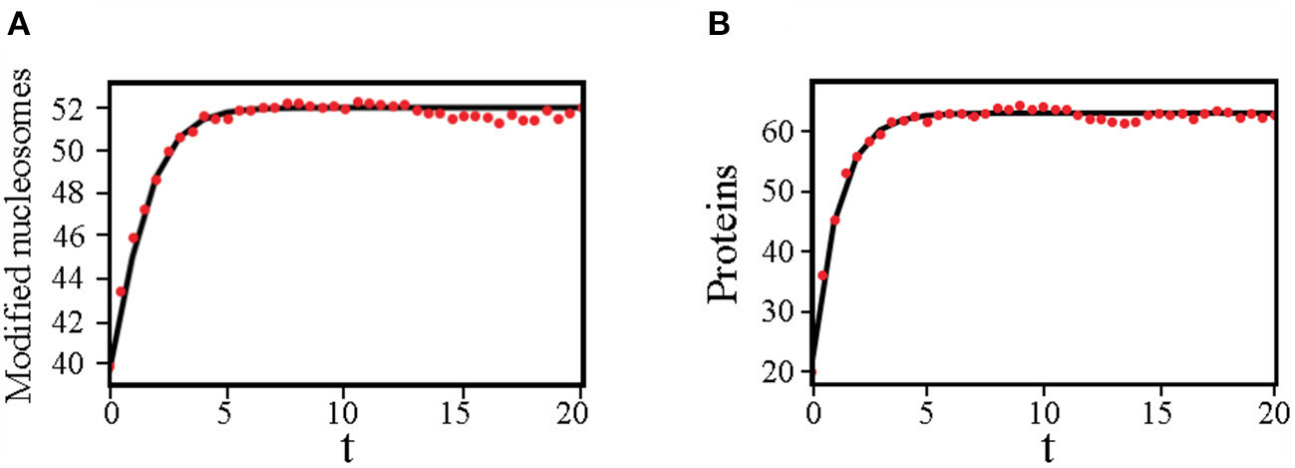
Dynamical trajectories determined from the variational approach agree well with stochastic simulations in favorable regimes. **(A)** Time evolution of the average number of modified nucleosomes computed using the variational method (black solid line) and stochastic simulations (red dots). **(B)** Time evolution of the average number of modified nucleosomes computed using the variational method (black solid line) and stochastic simulation (red dots). We used *q* = 10, *M* = 60, and set *c*_1_*p*_1_ = 0, *c*_0_*p*_0_ = 20, *c*_1_*t*_1_ = 0, *c*_1_*t*_0_ = 0.66 as the initial values when solving the deterministic equations (Equation 11).

We carried out stochastic simulations of the kinetic model using the Gillespie algorithm (Gillespie, 1977). Each plot shown in Figure 2 was obtained from averaging over 100 independent 10^5^-second-long simulations. These trajectories were initialized with random configurations, and the number of modified nucleosomes and protein molecules along each trajectory was recorded at every second. We then combined the values from all trajectories to estimate the steady state probability distributions, *P*_*ss*_. For the plots shown in Figure 3 we used *q* = 10 and set *n*_*x*_ = 40 and *n*_*p*_ = 20 at *t* = 0. 200 independent trajectories were performed to produce the average numbers recorded at every 0.5 s.

## 3. Theory

We reformulated the master equation describing the dynamical evolution of the kinetic network as an *imaginary* time Schrödinger equation

(4) $$\begin{array}{l} \partial_{t}|\Psi (t)\rangle =\Omega |\Psi (t)\rangle . \end{array}$$

The state vector $|\Psi (t)\rangle =\left(\begin{array}{c} \Psi_{1}(t)\\ \Psi_{0}(t) \end{array}\right)$ is a superposition of all possible configurations weighted with their corresponding probabilities such that $\Psi_{i}(t)=\sum_{n_{p},n_{x}}P_{i}(\{n_{p},n_{x}\};t)|n_{p},n_{x}\rangle$ for *i* = 0, 1. The two components correspond to the DNA state with regulatory proteins unbound (state 1) or bound (state 0), respectively. This reformulation makes use of a second quantization based method (the Doi-Peliti approach), which has been successfully employed in the study of reaction-diffusion processes (Lee and Cardy, 1995), gene switches (Sasai and Wolynes, 2003; Zhang and Wolynes, 2014), and other systems (Täuber, 2014). In previous work, we applied the Doi-Peliti approach to the epigenetic switch using operators that are a representation of the SU(2) algebra (Sood and Zhang, 2020). The SU(2) algebra allows us to treat the constraint of conservation of particle in number in a mathematically elegant and convenient way. When coupled to the self-activating gene, the stochastic Hamiltonian for the system described in Figure 1 is given by

(5) $$\begin{array}{l} \Omega =g\left(a_{p}^{†}-1\right)+k\left(a_{p}-a_{p}^{†}a_{p}\right)+s\left[J_{+}{\hat{n}}_{x}^{\underline{2}}-{\hat{n}}_{x}^{\underline{2}}{\hat{n}}_{y}\right]\\ \;+z\left[J_{-}{\hat{n}}_{y}^{\underline{2}}-{\hat{n}}_{y}^{\underline{2}}{\hat{n}}_{x}\right]+q\left[J_{-}-{\hat{n}}_{x}\right]+q\left[J_{+}-{\hat{n}}_{y}\right]\\ \;+\left(\begin{array}{cc} -h\left({\hat{n}}_{p},{\hat{n}}_{x}\right) & f\\ h\left({\hat{n}}_{p},{\hat{n}}_{x}\right) & -f \end{array}\right), \end{array}$$

where $g=\left(\begin{array}{cc} g_{1} & 0\\ 0 & g_{0} \end{array}\right),s=\left(\begin{array}{cc} s_{1}/N^{3} & 0\\ 0 & s_{0}/N^{3} \end{array}\right),z=\left(\begin{array}{cc} z/N^{3} & 0\\ 0 & z/N^{3} \end{array}\right),k=\left(\begin{array}{cc} k & 0\\ 0 & k \end{array}\right),q=\left(\begin{array}{cc} q/N & 0\\ 0 & q/N \end{array}\right)$, and $h\left({\hat{n}}_{p},{\hat{n}}_{x}\right)=\frac{{\hat{n}}_{p}\left({\hat{n}}_{p}-1\right)}{1+\text{exp}\left(-0.5\left({\hat{n}}_{x}-35\right)\right)}.$ The operator $a_{p}^{†}$ creates a protein molecule when it acts on a state, $a_{p}^{†}|n_{p},n_{x}\rangle =|n_{p}+1,n_{x}\rangle$, whereas *a*_*p*_ serves to remove a protein molecule when acting on the same state, *a*_*p*_ |*n*_*p*_, *n*_*x*_〉 = *n*_*p*_ |*n*_*p*_ − 1, *n*_*x*_〉. *J*_+_ converts an unmodified nucleosome to a modified one by acting on a state, *J*_+_ |*n*_*p*_, *n*_*x*_〉 = (*N* − *n*_*x*_) |*n*_*p*_, *n*_*x*_ + 1〉, while *J*_−_ acts to convert a modified nucleosome to an unmodified one, *J*_−_ |*n*_*p*_, *n*_*x*_〉 = *n*_*x*_ |*n*_*p*_, *n*_*x*_ − 1〉. ${\hat{n}}_{p}$ denotes the number operator, as its action on a ket gives the number of protein molecules, ${\hat{n}}_{p}|n_{p},n_{x}\rangle =n_{p}|n_{p},n_{x}\rangle$. In a similar fashion, ${\hat{n}}_{x}$ gives the number of modified nucleosomes when it acts on a ket, ${\hat{n}}_{x}|n_{p},n_{x}\rangle =n_{x}|n_{p},n_{x}\rangle$, and ${\hat{n}}_{y}$ gives the number of unmodified nucleosomes, ${\hat{n}}_{y}|n_{p},n_{x}\rangle =(N-n_{x})|n_{p},n_{x}\rangle$. *n*^2^ = *n*(*n* − 1) denotes the falling factorial.

Exact solutions to Equation (4) are difficult to obtain. Instead, we make use of an approximate, yet succinct and powerful, variational approach originally introduced by Eyink (Eyink, 1996; Alexander and Eyink, 1997). First, we realize that the imaginary time Schrödinger equation is equivalent to the functional variation of the following action Γ with respect to Φ, i.e., $\frac{\delta \Gamma }{\delta \Phi }=0$ for

(6) $$\begin{array}{l} \Gamma =\int \text{d}t\langle \Phi |\partial_{t}-\Omega |\Psi \rangle . \end{array}$$

By designing trial functions for Φ and Ψ parameterized with $\alpha_{L}=\alpha_{L}^{1},\alpha_{L}^{2},\cdots \;,\alpha_{L}^{K}$ and $\alpha_{R}=\alpha_{R}^{1},\alpha_{R}^{2},\cdots \;,\alpha_{R}^{K}$, minimizing the action leads to a set of ordinary differential equations,

(7) $$\begin{array}{l} {\sum_{l=1}^{K}\left[\langle \frac{\partial \Phi }{\partial \alpha_{L}^{m}}\rangle \frac{\partial \Psi }{\partial \alpha_{R}^{l}}\frac{\text{d}\alpha_{R}^{l}}{\text{d}t}-\langle \frac{\partial \Phi }{\partial \alpha_{L}^{m}}|\Omega |\Psi \rangle \right]}_{\alpha_{L}^{m}=0}=0, \end{array}$$

(8) $$\begin{array}{l} \text{for }m=1,\cdots \;,K. \end{array}$$

Also, we demand (to stay true to the probabilistic interpretation) 〈Φ(***α***_*L*_ = 0)〉Ψ(***α***_*R*_) = 1. The variational approach was first applied with great success to stochastic gene regulatory networks by Sasai and Wolynes (2003). In its original formulation, Poisson distributions were used as trial functions, with the Poisson mean being the variational parameter. Since protein molecules can be approximately treated as products of a birth-death process, the probability distribution to find *n*_*p*_ molecules should be Poisson at large *t* (Sasai and Wolynes, 2003). Furthermore, the stochastic Hamiltonian for genetic networks consists of only Bosonic operators, the coherent states of which correspond to Poisson distributions. In this work, we exploit the symmetry imposed on the system by particle number constraints to derive a new variational trial function for the chromatin switch. As shown in the Supplementary Material, an excellent candidate is the binomial distribution function since the coherent states for the SU(2) operators in our stochastic Hamiltonian are binomial (Fu and Sasaki, 1997, 1998). Taken together, we can thus use the following ansatz as variational functions for the coupled genetic and epigenetic switch

(9) $$\begin{array}{l} |\Psi \rangle =\left(\begin{array}{c} c_{1}\exp\left(p_{1}(a_{p}^{†}-1)\right){\left(1-\theta_{1}\right)}^{N}\exp\left(\frac{\theta_{1}}{1-\theta_{1}}J_{+}\right)|0,0\rangle \\ c_{0}\exp\left(p_{0}(a_{p}^{†}-1)\right){\left(1-\theta_{0}\right)}^{N}\exp\left(\frac{\theta_{0}}{1-\theta_{0}}J_{+}\right)|0,0\rangle \end{array}\right), \end{array}$$

and

(10) $$\begin{array}{l} \langle \Phi |=(\langle 0,0|e^{a_{p}}e^{J_{-}}\exp\left(\alpha_{1}+\lambda_{1}^{\left(p\right)}a_{p}+\lambda_{1}^{\left(x\right)}J_{-}\right)\\ \;\langle 0,0|e^{a_{p}}e^{J_{-}}\exp\left(\alpha_{0}+\lambda_{0}^{\left(p\right)}a_{p}+\lambda_{0}^{\left(x\right)}J_{-}\right)). \end{array}$$

The set of variational parameters is ***α***_*R*_ = {*c*_1_, *c*_0_, *p*_1_, *p*_0_, θ_1_, θ_0_}. Here *c*_1_(*c*_0_) represents the probability of the DNA being in state 1 (state 0), while *p*_1_(*p*_0_) and *Nθ*_1_(*Nθ*_0_) represent the mean number of proteins and modified nucleosomes when DNA is in state 1 (state 0). $\alpha_{L}=\left\{\alpha_{1},\alpha_{0},\lambda_{1}^{\left(p\right)},\lambda_{0}^{\left(p\right)}\lambda_{1}^{\left(x\right)},\lambda_{0}^{\left(x\right)}\right\}$ are the corresponding conjugate variables.

Plugging (10) and (9) into (7), we obtain the following set of variational equations

(11a) $$\begin{array}{l} \frac{\text{d}c_{1}}{\text{d}t}=c_{0}f-c_{1}{\langle h(n_{p},n_{x})\rangle }_{1} \end{array}$$

(11b) $$\begin{array}{l} \frac{\text{d}c_{0}}{\text{d}t}=-c_{0}f+c_{1}{\langle h(n_{p},n_{x})\rangle }_{1} \end{array}$$

(11c) $$\begin{array}{ll} c_{1}\frac{\text{d}p_{1}}{\text{d}t}+p_{1}\frac{\text{d}c_{1}}{\text{d}t} & =c_{1}g_{1}-c_{1}kp_{1}+c_{0}fp_{0}-c_{1}{\langle n_{p}h(n_{p},n_{x})\rangle }_{1} \end{array}$$

(11d) $$\begin{array}{ll} c_{0}\frac{\text{d}p_{0}}{\text{d}t}+p_{0}\frac{\text{d}c_{0}}{\text{d}t} & =c_{0}g_{0}-c_{0}kp_{0}-c_{0}fp_{0}+c_{1}{\langle n_{p}h(n_{p},n_{x})\rangle }_{1} \end{array}$$

(11e) $$\begin{array}{l} N\theta_{1}\frac{\text{d}c_{1}}{\text{d}t}+Nc_{1}\frac{\text{d}\theta_{1}}{\text{d}t}=c_{1}\left(\frac{s_{1}}{N^{3}}\right){\langle n_{x}^{\underline{2}}(N-n_{x})\rangle }_{1}\\ \;-c_{1}\left(\frac{z_{1}}{N^{3}}\right){\langle {\left(N-n_{x}\right)}^{\underline{2}}(n_{x})\rangle }_{1}\\ \;+c_{1}\frac{q}{N}(-{\langle n_{x}\rangle }_{1}+{\langle N-n_{x}\rangle }_{1})\\ \;+c_{0}fN\theta_{0}-c_{1}{\langle n_{x}h(n_{p},n_{x})\rangle }_{1} \end{array}$$

(11f) $$\begin{array}{l} N\theta_{0}\frac{\text{d}c_{0}}{\text{d}t}+Nc_{0}\frac{\text{d}\theta_{0}}{\text{d}t}=c_{0}\left(\frac{s_{0}}{N^{3}}\right){\langle n_{x}^{\underline{2}}(N-n_{x})\rangle }_{0}\\ \;-c_{0}\left(\frac{z_{0}}{N^{3}}\right){\langle {\left(N-n_{x}\right)}^{\underline{2}}(n_{x})\rangle }_{0}\\ \;+c_{0}\frac{q}{N}(-{\langle n_{x}\rangle }_{0}+{\langle N-n_{x}\rangle }_{0})-c_{0}fN\theta_{0}\\ \;-c_{1}{\langle n_{x}h(n_{p},n_{x})\rangle }_{1}. \end{array}$$

The angular brackets represent ensemble averaging over protein numbers and modified nucleosomes, i.e., ${\langle \cdot \rangle }_{i}=\underset{n_{p},n_{x}}{\sum }\cdot \frac{e^{-p_{i}}}{n_{p}!}p_{i}^{n_{p}}\left(\begin{array}{c} N\\ n_{x} \end{array}\right)\theta_{k}^{n_{x}}{\left(1-\theta_{k}\right)}^{N-n_{x}}$. We also make the simplifying approximation for the average binding rate as $\langle h\left(n_{p},n_{x}\right)\rangle =\langle \frac{n_{p}\left(n_{p}-1\right)}{1+\text{exp}\left(0.5\left(n_{x}-35\right)\right)}\rangle \approx \frac{{\langle n}_{p}(n_{p}-1)\rangle }{1+\text{exp}\left(0.5\left({\langle n}_{x}\rangle -35\right)\right)}$. Numerical integration of Equation (11) yields the time evolution of the variational parameters ***α***_*R*_, from which the probability distributions can be determined using Equation (9).

We solved Equation (11) using scipy.integrate.odeint() module in python with a time step of 0.01 s. The initial conditions were varied and individual trajectories were integrated for 10^5^ s till convergence to obtain the steady state results.

## 4. Results

Using the variational equations, we studied the dependence of steady-state solutions on the rate of noisy histone mark modification, *q*. For comparison, we carried out stochastic simulations of the kinetic network using the Gillespie algorithm (Gillespie, 1977) at selected *q*-values. The noisy modification rate and, in particular, its relative value to the rate for recruited conversions is an important parameter for cell differentiation. For example, recruited conversions arise due to the diffusion of histone-modifying enzymes from modified nucleosomes to the nearby unmodified ones. The more open chromatin conformation seen in stem cells with larger inter-nucleosome distances (Gaspar-Maia et al., 2011; Mas et al., 2018) will, therefore, suppress recruited conversions in favor of the noisy ones. As cells differentiate, chromatin will become more compact, and the importance of noisy conversions will decline. Previous studies of isolated epigenetic switches (Dodd et al., 2007; Micheelsen et al., 2010; Sood and Zhang, 2020) also found *q* as an important parameter that controls the onset and maintenance of bistability in the epigenetic switch.

In Figure 2, we show the probability distributions obtained from stochastic simulations and from the variational approach at *q* = 100, 10, and 0.5. We notice that the Binomial ansatz introduced in the *Theory* section captures the distribution for the number of modified nucleosomes with quantitative accuracy (Figures 2A–C). The Poissonian ansatz also performs well for the distribution of protein numbers at small and medium *q* values, though deviations from stochastic simulations are apparent at large *q* (Figures 2D–F). The inconsistency between the two distributions in that regime is mainly due to underestimating the population of intermediate states that bridge the high and low gene expression values by the variation method.

In addition to steady-state solutions, the time evolution of observables, such as the mean number of proteins and modified nucleosomes, can be determined using the variational approach as well. As shown in Figure 3, in parameter regimes where the effect of fluctuations is not too drastic, the dynamical trajectories determined using Equation (11) are in quantitative agreements with those computed using stochastic simulations.

Given its reasonable performance, we next applied the variational approach to study the network model's steady-state behavior at a broader range of *q*-values. As already mentioned, *q* is an important variable that might be tuned along the developmental axis for cell differentiation. For large *q* values, chromatin stabilizes in an undecided state with roughly half the nucleosomes modified (active) and the other half carrying no modification (repressive). The corresponding protein expression is noisy with a broad probability distribution. Stochastic simulations further support a significant mixing between “on” and “off” gene states, and an unambiguous assignment of either state is not warranted (Figure 2D). When the value for *q* is quenched, we observe the emergence of a coherent epigenetic state along with coherent gene expression. Therefore, both switches are turned on and the combined system exhibits a single attractor. At even lower values of *q*, both the epigenetic and gene switch exhibit bistability.

**Figure 4:**
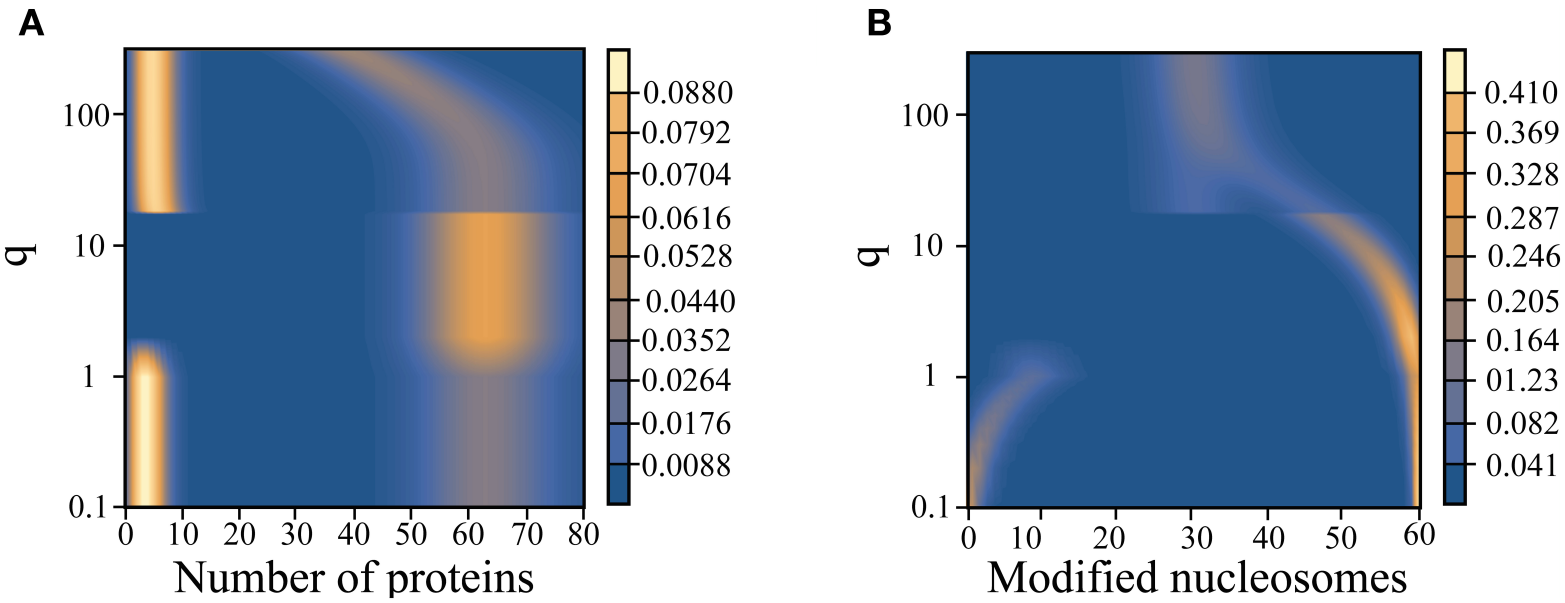
Variation of the steady state probability distribution for the number of proteins **(A)** and modified nucleosomes **(B)** as a function of the noisy histone modification rate, *q*.

We note that the chromatin state changes described above differs from that of an isolated epigenetic switch studied previously (Sood and Zhang, 2020). There, we saw a shift from a unimodal probability distribution indicating an equal admixture of modified and unmodified nucleosomes to a symmetric bimodal probability distribution as the value for *q* is quenched. The appearance of a single coherent epigenetic state in Figure 4 is a result of the coupling with the gene switch in our model, which breaks the symmetry between active and repressive chromatin states. The coupling works both ways. In an isolated gene switch, a single state with high gene expression is not expected either. Modulating the kinetics of TF binding to the promoter only resolves a broad probability distribution exhibiting no coherent gene expression to a bistable state with high and low levels of gene expression (Walczak et al., 2005a).

## 5. Discussion

We introduced a kinetic model that couples a genetic network with an epigenetic switch to study the combined role of transcription factors and histone modifications in regulating gene expression. An approximation scheme based on the variational approach was further developed to obtain steady-state solutions. This method is unencumbered by the complexity associated with numerical simulations and more detailed analytical calculations. It would be a useful tool for exploratory studies of the parameter space and identifying regions of interest. While we focused our analysis on a single gene, the variational method can be relatively easily generalized to networks with multiple interacting genetic and epigenetic switches that provide more realistic modeling of stem cell differentiation (Zhang and Wolynes, 2014).

We explored the behavior of the network model across a wide range of parameters. Our model exhibits a poised state for the gene switch at high *q*, where the chromatin system contains an equal admixture of modified and unmodified nucleosomes. The network in this parameter regime appears to qualitatively capture the behavior of chromatin and gene expression in undifferentiated stem cells. In particular, stem cells are known to exhibit bivalent chromatin with both activating and repressive marks (Bernstein et al., 2006; Vastenhouw and Schier, 2012) and noisy gene expression profiles (Kar et al., 2017). We point out that the exact definition of bivalent chromatin remains controversial, and multiple mechanisms have been proposed for its formation (Azuara et al., 2006; Sneppen and Ringrose, 2019; Lim and Meshorer, 2020). Additional studies are needed to determine whether the stochastic conversion observed here is the key driver for the observed chromatin bivalency.

Upon quenching *q*, the gene is activated along with a concomitant resolution of the chromatin state. The coupling between the two switches reinforces the stability of the active state and can lead to more robust upregulation of gene expression upon cell differentiation. It also ensures that the genetic and epigenetic switches are always in sync. We observe at most two steady states representing active chromatin with high gene expression and repressive chromatin with low gene expression. We note that the inactive state only becomes stable at minimal *q* values, arguing for strong noise suppression for gene silencing. Its limited stability may explain the presence of DNA methylation on top of histone modifications to safeguard the silent state against perturbations that might arise from fluctuation in protein concentration or histone marks during cell division.

The strong dependence of the landscape tomography on *q* shown in Figure 4 suggests that the histone modification rate may act like a knob to be tuned along the developmental axis to facilitate cellular differentiation. Of course, the presented landscape is probably too crude a simplification to be termed the Waddington landscape since many additional factors that contribute to the stability of gene expression patterns could be varied along the developmental axis as well.

In favorable regimes, the variational approach produces results of quantitative accuracy. The discrepancy between the probability distribution obtained from stochastic simulations and the variational method in the high *q* region can be attributed to the fact that the Poisson ansatz does not sufficiently account for the variance and the effect of fluctuations which become increasingly important as the value for *q* increases. This situation can be remedied by going beyond the Poisson ansatz, and utilizing the superposition ansatz as described in Ohkubo (2008). Mathematically, this would mean to modify our ansatz as follows,

(12) $$\begin{array}{l} |\Psi \rangle \\ =\left(\begin{array}{c} \int_{0}^{\infty }\text{d}p_{1}\;F(p_{1};\{\lambda_{j}^{\left(1\right)}\})\;c_{1}\exp\left(p_{1}(a_{p}^{†}-1)\right){\left(1-\theta_{1}\right)}^{N}\exp\left(\frac{\theta_{1}}{1-\theta_{1}}J_{+}\right)|0,0\rangle \\ \int_{0}^{\infty }\text{d}p_{0}\;F(p_{0};\{\lambda_{j}^{\left(0\right)}\})\;c_{0}\exp\left(p_{0}(a_{p}^{†}-1)\right){\left(1-\theta_{0}\right)}^{N}\exp\left(\frac{\theta_{0}}{1-\theta_{0}}J_{+}\right)|0,0\rangle \end{array}\right). \end{array}$$

This new “superposition ansatz” is constructed by the superposition of the coherent states (i.e., Poisson distribution) as defined in (12), where now F serves as the variational function. Hence, the real probability distribution is obtained by the superposition of the Poisson distributions of mean *p*_*i*_ weighed by the distribution F with parameters $\left\{\lambda_{j}^{\left(i\right)}\right\}$. We anticipate that doing so can not only improve the agreement between theory and simulation but can in principle allow for the computation of time evolution of other interesting quantities such as variance, and covariance in addition to means. However, in general the choice of an appropriate F is a non-trivial problem, and thus has been avoided in this text in favor of a clearer exposition. The choice of appropriate variational functions can be guided by the work done on exact solutions of the master equations of genetic switches (Hornos et al., 2005; Shahrezaei and Swain, 2008; Ramos et al., 2011).

## Data Availability Statement

The original contributions presented in the study are included in the article/Supplementary Material, further inquiries can be directed to the corresponding author/s.

## Author Contributions

AS and BZ conceived and designed the work, interpreted the results, and wrote the manuscript. AS carried out computer implementation and data analysis. BZ supervised the project. All authors contributed to the article and approved the submitted version.

## Conflict of Interest

The authors declare that the research was conducted in the absence of any commercial or financial relationships that could be construed as a potential conflict of interest.
