# Supplementary material for: Quantifying the Stability of Coupled Genetic and Epigenetic Switches With Variational Methods
Source: Front Genet. 2021 Jan 22;11:636724. doi: 10.3389/fgene.2020.636724 (PMC7862759; doi:10.3389/fgene.2020.636724)
Supplement: Supplementary file 1 [file Presentation_1.PDF]

Supplementary Material

DERIVATION OF TRIAL FUNCTION FOR THE EPIGENETIC SWITCH

The connection between multinomial probability distributions and SU(n) algebras has been studied from an algebraic perspective, and we refer interested readers to the literature (Fu and Sasaki, 1997, 1998). Here we present an alternate derivation that is perhaps simpler and more intuitive. Keeping with standard literature conventions, we start with the trial function in the Poisson ansatz (Ohkubo, 2008) for a two particle system,

$$|\Psi(t)\rangle = \sum_{\{n_x,n_y\}} P(\{n_x, n_y\}; t) |n_x, n_y\rangle . \tag{S1}$$

with $|n_x, n_y\rangle = (a_x^\dagger)^{n_x}(a_y^\dagger)^{n_y} |0, 0\rangle$, and $P(\{n_x, n_y\}; t)$ being the product of two independent Poisson distributions in the Poisson ansatz,

$$P(\{n_x, n_y\}; t) = \frac{e^{-x}x^{n_x}}{n_x!}\frac{e^{-y}y^{n_y}}{n_y!}. \tag{S2}$$

Here x, y are parameters of the Poisson distribution, and we can treat them as time dependent variational parameters. One observes that in the Poisson ansatz, $|\Psi\rangle = |\psi_x\rangle \otimes |\psi_y\rangle$, where one frequently encounters the abbreviated form for $|\psi_i\rangle = \exp\Big(\mu_i(a_i^\dagger - 1)\Big) |0_i\rangle$. In order to proceed, we impose $P(\{n_x, n_y\}; t) = \frac{e^{-x}x^{n_x}}{n_x!}\frac{e^{-y}y^{n_y}}{n_y!}\delta_{n_x+n_y,N}$, and normalized by $\sum \frac{e^{-x}x^{n_x}}{n_x!}\frac{e^{-y}y^{n_y}}{n_y!}\delta_{n_x+n_y,N} = 1$. We then note $x + y = N$, $n_x \equiv n$, and $n_y = N - n_x$. Introducing a new parameter θ such that $x = N\theta$, and $y = N(1 - \theta)$, one arrives at, $P(\{\theta, N\}; t) = \binom{N}{n_x}\theta^{n_x}(1 - \theta)^{N-n_x}$, which is the Binomial probability distribution. Thus, the variational ansatz for two-particle system with a constraint $N = n_x + n_y$ is reduced to,

$$|\Psi(t)\rangle = \sum_n \mathrm{Bin}(\theta, N; t) |n\rangle , \tag{S3}$$

where θ is the appropriate time-dependent variational parameter. Any arbitrary state is now, $|n_x, N - n_x\rangle = (a_x^\dagger)^{n_x}(a_y^\dagger)^{N-n_x} |0, 0\rangle \equiv \frac{J_+^n}{N^{\underline{n}}} |0\rangle$, where $x^{\underline{n}} = x(x - 1)(x - 2)\ldots(x - n + 1)$ denotes the falling factorial. This allows us to write the binomial varitional ansatz more succinctly as,

$$|\Psi\rangle = (1 - \theta)^N \exp\left(\frac{\theta}{1 - \theta}J_+\right) |0\rangle . \tag{S4}$$

The action of the operator J_+ on a ket, and its algebraic properties are discussed in greater detail in the literature (Sood and Zhang, 2020).

REFERENCES

Fu, H.-C. and Sasaki, R. (1997). Negative binomial and multinomial states: Probability distributions and coherent states. *Journal of Mathematical Physics* 38, 3968–3987

Fu, H.-C. and Sasaki, R. (1998). Probability distributions and coherent states of , and algebras. *Journal of Physics A: Mathematical and General* 31, 901–925

Ohkubo, J. (2008). Approximation scheme for master equations: Variational approach to multivariate case. *The Journal of Chemical Physics* 129, 044108

Sood, A. and Zhang, B. (2020). Quantifying epigenetic stability with minimum action paths. *Phys. Rev. E* 101, 062409
